# An Intraoperative EEG Biomarker for Postoperative Delirium Predicting Based on Interpretable Deep Learning Framework

**DOI:** 10.1002/mco2.70980

**Published:** 2026-09-06

**Authors:** Yinuo Zhang, Yan Zhu, Xinxin Zhang, Xinke Shen, Xuemiao Tang, Zhihong Lu, Chong Lei, Mengyu Li, Hailong Dong, Zhichao Liang, Quanying Liu, Guangchao Zhao

**Affiliations:** ^1^ Department of Biomedical Engineering Southern University of Science and Technology Shenzhen Guangdong China; ^2^ Department of Anesthesiology and Perioperative Medicine Xijing Hospital The Fourth Military Medical University Xi'an China; ^3^ Key Laboratory of Anesthesiology (The Fourth Military Medical University) Ministry of Education Xi'an China; ^4^ Shaanxi Provincial Clinical Research Center for Anesthesiology Medicine Xi'an China; ^5^ Department of Anesthesiology The Third People's Hospital of Chengdu Chengdu Sichuan China; ^6^ Center for Neurocognition and Social Behavior Artificial Intelligence Research Institute Shenzhen University of Advanced Technology Shenzhen Guangdong China

**Keywords:** interpretable deep learning framework, multichannel EEG recording, postoperative delirium, spatiotemporal convolutional network

## Abstract

Postoperative delirium (POD) is a common complication in older surgical patients and substantially worsens clinical outcomes, yet existing intraoperative electroencephalography (EEG) monitoring tools lack spatial and temporal specificity, creating a need for interpretable biomarkers. We prospectively analyzed 32‐channel intraoperative EEG from 71 patients aged ≥ 60 undergoing noncardiac surgery, trained an interpretable spatiotemporal convolutional network (ST‐CN), derived a best temporal filter (BTF), and evaluated model performance with region‐specific tests and independent external validation. The ST‐CN classified POD with 97.52% accuracy and an ROC of 0.996. The BTF alone discriminated POD with an AUC of 0.911 and achieved 85.12% accuracy using frontal EEG alone. It captured a distinct 2–12 Hz (δ–θ–α) oscillation in a spindle‐like envelope, which occurred at a significantly higher rate in POD patients (4.62 ± 0.15 vs. 3.88 ± 0.13 waves/min in the frontal region) and differed in central frequency and spectral power. Independent external validation further confirmed robust generalizability, with frontal EEG achieving 89.01% accuracy and an AUC of 0.950. This framework enables accurate, interpretable POD risk stratification and identifies a reproducible frontal EEG biomarker, supporting objective intraoperative early warning and individualized perioperative care.

## Graphical abstract

An interpretable intraoperative EEG framework predicts postoperative delirium (POD) in older surgical patients. A data‐driven best temporal filter (BTF) reveals frontal sensitivity and captures a distinctive 2–12 Hz (δ–θ–α) spindle‐like target wave occurring more frequently in POD patients, enabling event‐quantifiable intraoperative monitoring and individualized perioperative care

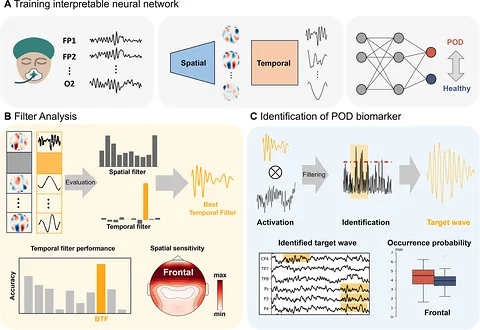

## 1 Introduction

Postoperative delirium (POD) is a prevalent acute perioperative neurocognitive complication characterized by conscious disturbances, cognitive decline, and abnormal perceptions, and so forth [1, 2, 3]. The POD incidences have been shown to prolong hospitalization duration, intensive care unit (ICU) stay, and duration of mechanical ventilation, while further increasing the risk of postoperative mortality and long‐term cognitive impairment [4, 5, 6]. Therefore, the development of novel techniques for POD early warning to optimize the individualized perioperative management strategies is of paramount clinical significance in the prevention of perioperative neurocognitive disorders and the improvement of patient outcomes.

Most risk assessment for POD in current studies encompasses comprehensive predictive models based on perioperative biomarkers reflecting multiorgan function, including blood gas analysis and oxygenation indices, inflammatory markers, hepatic and renal function indicators, and neurology‐related biomarkers [7, 8]. Additionally, direct multimodal evaluations of brain functional state, such as electroencephalography (EEG) and functional magnetic resonance imaging (fMRI), are also utilized [7, 8]. However, these models have several limitations in clinical practice for POD prediction. In recent years, the analysis of dynamic EEG characteristics across the entire perioperative period using multichannel EEG recordings has emerged as a critical avenue for POD prediction and susceptibility identification. For example, the reduced intraoperative frontal α power has been associated with an increased risk of POD [9, 10], and POD patients demonstrate abnormalities in frontoparietal network connectivity [11]. The available evidence suggests that employing interpretable deep learning frameworks to simultaneously integrate both temporal and spatial activation features from intraoperative EEG could potentially capture more precise POD‐specific EEG events, which would facilitate the development of early prediction models for POD with higher reliability and validity.

Here, we leverage a spatiotemporal convolutional network (ST‐CN) based on intraoperative EEG signals to develop an interpretable, data‐driven binary classification framework for POD early warning. The ST‐CN, which cascades spatial and temporal convolutional layers, adaptively extracts multiscale patterns and captures complex spatiotemporal dependencies inherent to neural signals [12]. A key advantage is interpretability: learned spatial and temporal filters provide physiologically meaningful representations. Beyond conventional prespecifying of frequency bands, the ST‐CN optimizes feature combinations in a data‐driven manner, reducing hand‐crafted bias and enhancing individual adaptability that support improved predictive performance and cross‐population generalizability [13, 14].

**FIGURE 1:**
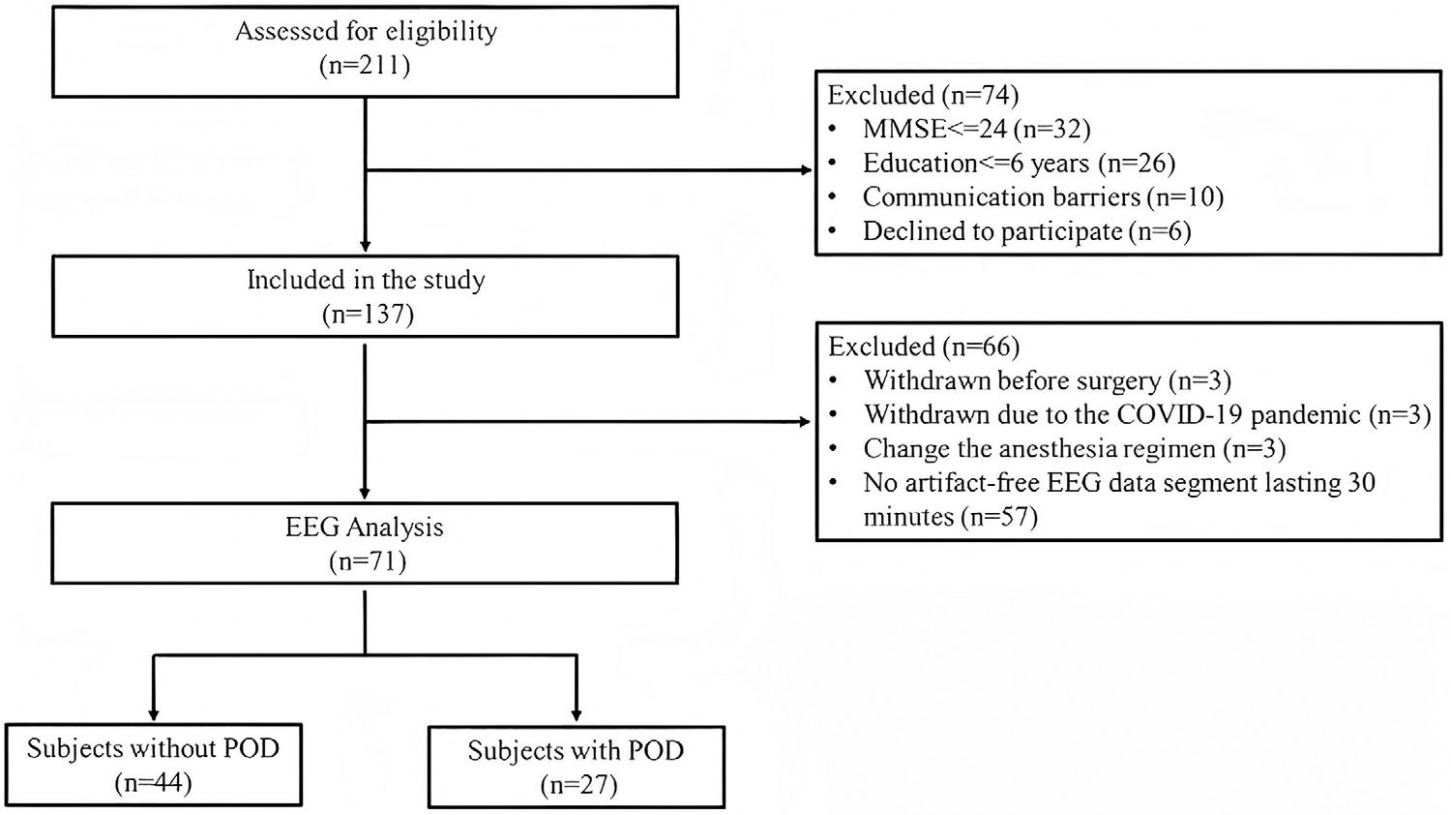
Flow chart of experimental design.

In this study, we constructed a novel early warning model for POD using 32‐channel intraoperative EEG data obtained from elderly patients undergoing noncardiac surgery in a single‐center prospective observational cohort study (Figure 1). The model achieved a classification accuracy of 97.52%. Through training, the ST‐CN identified a single best temporal filter (BTF) that alone enabled robust prediction and localized the effective predictive sampling scope to the frontal cortex, thereby improving operational feasibility in clinical practice. The electrophysiological signature captured by this BTF primarily presents as a broad‐band 2–12 Hz (encompassing δ, θ, and α bands) oscillatory coupling entrained within spindle‐like waveform envelopes. This pattern occurred at a significantly higher density in the intraoperative EEG of patients with POD. Collectively, these findings suggest that this newly identified EEG signature may serve as an interpretable neurophysiological indicator for early diagnosis and guidance of postoperative intervention in POD.

## 2 Results

### 2.1 Demographic Data

Of the 211 patients assessed for eligibility, 74 were excluded (32 with MMSE ≤ 24; 26 with education ≤ 6 years; 10 with communication barriers; six who declined participation), resulting in 137 patients initially included in the study. Subsequently, 66 patients were withdrawn (three before surgery; three due to the COVID‐19 pandemic; three due to changes in anesthesia protocol; and 57 with no artifact‐free 30‐min EEG data), leaving 71 patients for final analysis (27 with POD, 44 without). The incidence of POD was 38.02% (Figure 1). The demographics, clinical characteristics, and anesthetic usage of enrolled patients were summarized in Table 1. There were no significant differences in age, sex, BMI, MMSE score, or ASA physical status between the non‐POD and POD groups. However, patients who developed POD showed a lower level of education. Intraoperatively, the two groups exhibited similar duration of general anesthesia, surgery, and comparable administration rates of most intravenous anesthetics. No significant differences were observed in the length of stay in the PACU or the hospital between these two groups. Given that different types of surgeries may have a significant impact on the incidence of POD, we have therefore detailed the specific surgical information of the patients enrolled in this study (see in Table S1).

**TABLE 1:** Comparison of demographic and perioperative data between POD and non‐POD patients.

Variable	Non‐POD, *n* = 44	POD, *n* = 27	*p* value
**Age, years**	67.00 (64.25, 71.00)	68.00 (63.00, 71.00)	0.8991
**Sex, *n* (%)**			0.5922
Female	9 (20.45)	7 (25.93)	
Male	35 (79.55)	20 (74.07)	
**Education, years**	9.00 (9.00, 12.00)	9.00 (6.00, 12.00)	0.0295*
**ASA physical status**			0.5043
II/III	37/7	21/6	
%	84.09/15.91	77.78/22.22	
**BMI, kg/m^2^**	24.43 ± 3.28	22.99 ± 3.07	0.0700
**MMSE score**	28.00 (27.00, 29.00)	28.00 (26.00, 29.00)	0.2162
**Intraoperative factors**			
Duration of surgery (min)	215.00 (164.80, 280.50)	206.00 (161.00, 339.00)	0.9836
Duration of anesthesia (min)	258.00 (211.80, 321.80)	252.00 (208.00, 399.00)	0.8346
Estimated blood loss (mL)	100.00 (50.00, 200.00)	150.00 (50.00, 400.00)	0.0746
Blood transfusion, *n* (%)	3 (6.82)	3 (11.11)	0.5278
PACU length of stay (min)	55.00 (41.25, 68.75)	55.00 (45.00, 65.00)	0.7296
Length of hospital stay (days)	12.00 (9.00, 19.00)	11.50 (9.00, 15.75)	0.8154

Data are presented as the mean (standard deviation), median (25%–75%), or number of patients (%). All data were compared using the unpaired *t*‐test, Mann–Whitney *U*‐test, or *χ* ^2^ test, as appropriate. *p < 0.05.

Abbreviations: ASA, American Society of Anesthesiologists; BMI, body mass index; MMSE, Mini‐Mental State Examination; PACU, postanesthesia care unit; POD, postoperative delirium.

Furthermore, we performed an additional external validation based on an independent dataset comprising 13 previously unseen patients (five with POD). This dataset was entirely excluded from model development and hyperparameter tuning, thereby providing a stringent assessment of cross‐dataset generalizability. Ethics approval and registration details for both cohorts are provided in the Ethics approval section.

### 2.2 POD Identification Based on the Spatiotemporal Convolutional Network Model

**FIGURE 2:**
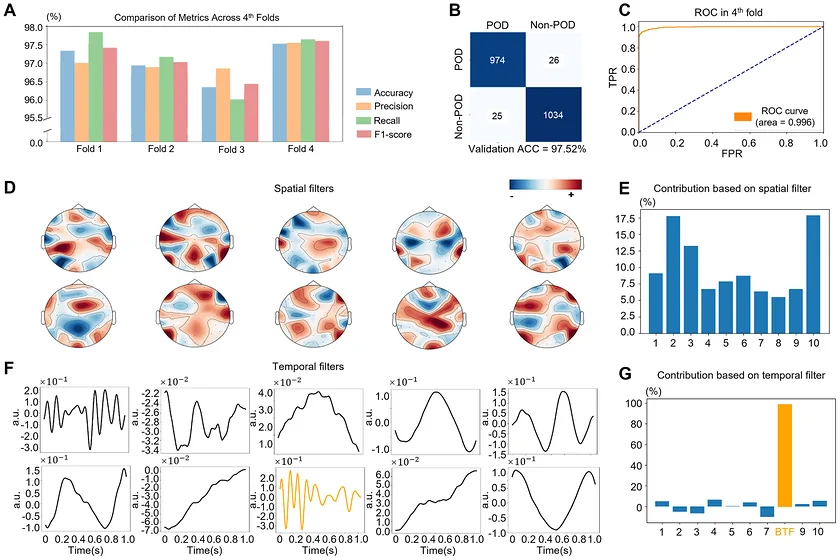
Classification performance and learned spatial/temporal filters of the spatiotemporal convolution model. (A) Classification performance of the model during fourfold cross‐validation. The fourth fold, which demonstrates more stable overall performance, is selected as the primary subject for subsequent analyses. (B) Confusion matrix of the Fold 4 model on the test set, showing a classification accuracy of 97.52%. (C) ROC curve of the Fold 4 model, with an AUC value of 0.996. (D) Visualization of the 10 spatial filters on the brain topography map, where red indicates activation and blue indicates suppression. (E) Normalized contribution of each spatial filter to classification, calculated using the mask method. (F) Visualization of the 10 temporal filters across the time series, where each window captures 1‐s information. (G) Normalized contribution of each temporal filter to classification, calculated using the mask method. The eighth temporal filter shows the highest contribution and is identified as the best filter (highlighted in orange).

Here, we developed an ST‐CN to classify POD and non‐POD conditions based on multichannels intraoperative EEG data. The EEG signals were segmented into 10‐s epochs, each sampled at 250 Hz to ensure temporal consistency across inputs. To assess the model's performance, we employed fourfold cross‐validation. The results demonstrated that the model exhibited exceptional and stable performance across the test sets, yielding the following metrics: Accuracy = 97.03% ± 0.44%, Precision = 97.03% ± 0.28%, Recall = 97.17% ± 0.70%, and F1‐score = 97.12% ± 0.44% (Figure 2A). Among the different folds, the model from Fold 4 was selected due to its superior overall performance, achieving an accuracy of 97.52% and an ROC value of 0.996 on the test set (Figure 2B,C, and the performance of the remaining folds is detailed in Figures S1–S3).

Owing to the distinctive architecture of the ST‐CN, we conducted an in‐depth interpretability analysis of both its spatial and temporal convolutional layers. The spatial convolutional filters function as spatial activation maps, while the temporal filters capture salient sequential dynamics within the EEG data (Figure 2D–F). To identify the spatiotemporal features most influential in discriminating between POD and non‐POD conditions, we applied the ablation protocol outlined in 2.7 Filter analysis. Our analysis revealed one particular temporal filter that contributed most significantly to model performance (Figure 2G). Given its critical role, we designated this filter as the “Best Temporal Filter” for distinguishing POD from non‐POD individuals.

### 2.3 Best Temporal Filter Captures an EEG Signature of POD Individuals

**FIGURE 3:**
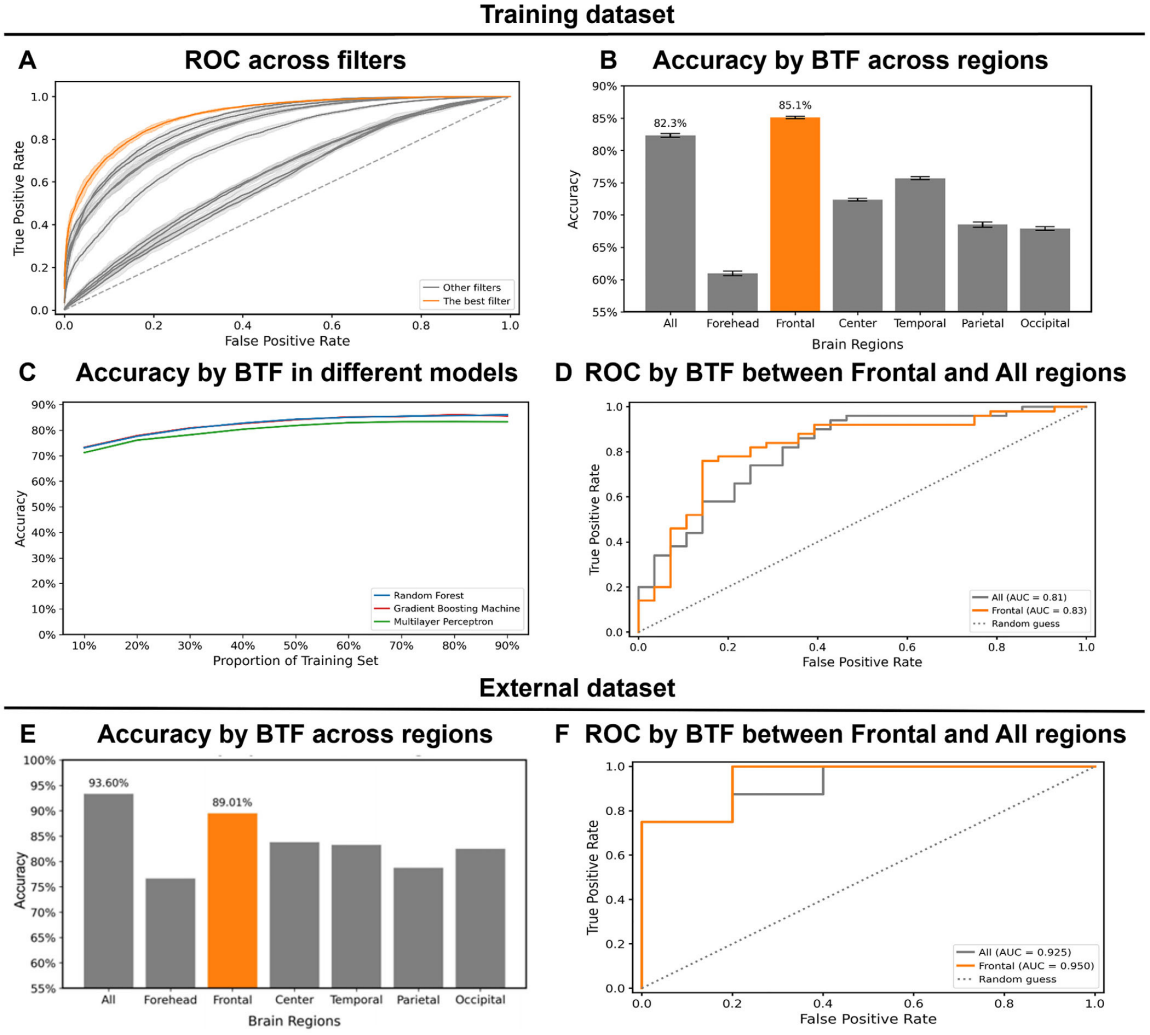
Reliability and generalizability of the learned best temporal filter. (A) The 10 temporal filters were applied to EEG signals as feature extractors. After dimensionality reduction, a random forest classifier was used to assess their performance. BTF exhibited the highest classification accuracy compared to the other temporal filters. (B) Classification of POD and non‐POD signals using the BTF and a random forest model applied to EEG signals from individual brain regions. The frontal region demonstrated the highest classification accuracy (85.32%). (C) Performance comparison of random forest (blue), gradient boosting machine (green), and multilayer perceptron (red) trained on datasets with varying proportions of training samples (10%–90%). All models showed stable classification performance, achieving over 80% accuracy with only 30% of the training data. (D) Features extracted using the BTF effectively discriminated between POD and non‐POD patients, achieving an AUC of 0.813. When using EEG signals from the frontal region, the classification performance improved further, reaching an AUC of 0.825. (E) Classification of POD and non‐POD signals using the BTF and a random forest model applied to EEG signals from individual brain regions in an external dataset. The All‐region signals demonstrated the highest classification accuracy (93.60%), followed by the frontal region (89.01%), indicating strong generalizability of the BTF to independent external data. (F) Features extracted using the BTF effectively discriminated between POD and non‐POD patients in an external dataset, achieving an AUC of 0.925. When using EEG signals from the frontal region, the classification performance improved further, reaching an AUC of 0.950.

To systematically evaluate the predictive performance of BTF for POD, we conducted a controlled experiment. In this study, we used the BTF and several candidate temporal filter as independent feature extractors. After applying principal component analysis for dimensionality reduction, we input the extracted features into a random forest classifier for binary classification, maintaining a training‐to‐test set ratio of 1:1. The ROC analysis revealed that the BTF achieved the highest discriminative performance (AUC = 0.911 ± 0.001), significantly outperforming the average performance of the other filters (AUC = 0.747 ± 0.004; Figure 3A).

We then investigated the spatial specificity of the BTF, which revealed its particular advantage in the frontal region. When we extracted features from individual brain regions and evaluated them through 10‐fold cross‐validation, the use of frontal EEG signals alone yielded an average classification accuracy of 85.11% ± 0.18%. This result substantially exceeded the accuracy from other individual regions and also surpassed the performance achieved with whole‐brain integration (82.32% ± 0.29%; Figure 3B). These findings are consistent with previous studies indicating that aberrant frontal α rhythms play a critical role in the pathophysiology of POD [15, 16].

Since most operating rooms use two‐channel or four‐channel frontal EEG monitors instead of 32‐channel systems, we further conducted additional analyses to evaluate the feasibility of implementing the BTF using standard low‐channel frontal EEG monitors. Initially, we verified the classification stability across varying training data proportions (10%–90%) and used multiple machine learning architectures, including random forest, gradient boosting machine, and multilayer perceptron. Even when using only 10% of the data for training, all models achieved accuracies above 70%. Performance exceeded 80% when the training proportion reached 50%, highlighting a notable sample efficiency (Figure 3C). Meanwhile, we constructed a classification model based on subject‐level statistical features (mean, standard deviation, extrema, and skewness). While integrating whole‐brain features yielded an AUC of 0.813, using frontal‐specific features improved diagnostic efficacy to an AUC of 0.825 (Figure 3D). Together, this hierarchical analytical strategy confirms the potential of the BTF for clinical application across both micro (EEG‐segment) and macro (patient‐level) scales.

Furthermore, to specify which frontal electrodes are essential for POD prediction, we assessed the classification accuracy of the BTF model using three different frontal electrode configurations: two‐channel (Group 1: FP1, FP2), four‐channel (Group 2: FP1, FP2, F7, F8), and five‐channel (Group 3: FP1, FP2, Fz, F3, F4). All analyses were performed using the same model parameters, preprocessing pipeline, and training procedures as in the primary study (Figure S4). The results demonstrated that the BTF model achieved classification accuracies of 61.7%, 69.9%, and 72.0% for the three frontal electrode configurations, respectively. While the two‐channel configuration (Group 1: FP1, FP2) showed relatively modest performance, the predictive accuracy improved substantially with increasing electrode density. Notably, when using a five‐channel frontal montage (Group 3: FP1, FP2, Fz, F3, F4), the BTF model achieved satisfactory POD prediction performance (ACC = 72.0%), suggesting the potential generalizability of this approach for clinical portable EEG recording systems. In future work, we will further explore optimized configurations with even fewer frontal channels tailored to clinical practical requirements, aiming to identify region‐specific and sensitive biomarkers.

Finally, to assess the generalizability of the learned BTF, we performed an external validation using an independent EEG dataset. Using the same BTF and classification pipeline, we evaluated region‐specific and whole‐brain EEG signals without retraining the temporal filter. In the external dataset, classification based on BTF‐convolved signals showed consistently high performance across brain regions, with the All‐region signals achieving the highest classification accuracy (93.60%), followed by the frontal region (89.01%; Figure 3E). Consistent with these accuracy results, ROC analysis on the external dataset further confirmed the strong discriminative capability of the BTF. Using All‐region EEG signals, the model achieved an AUC of 0.925, while frontal‐region EEG signals achieved an AUC of 0.950 (Figure 3F). These results demonstrate that the EEG signature captured by the BTF generalizes robustly to independent external data, supporting its potential translational applicability across cohorts and recording settings.

### 2.4 A Novel EEG Waveform as a Potential Biomarker for POD Early Warning

**FIGURE 4:**
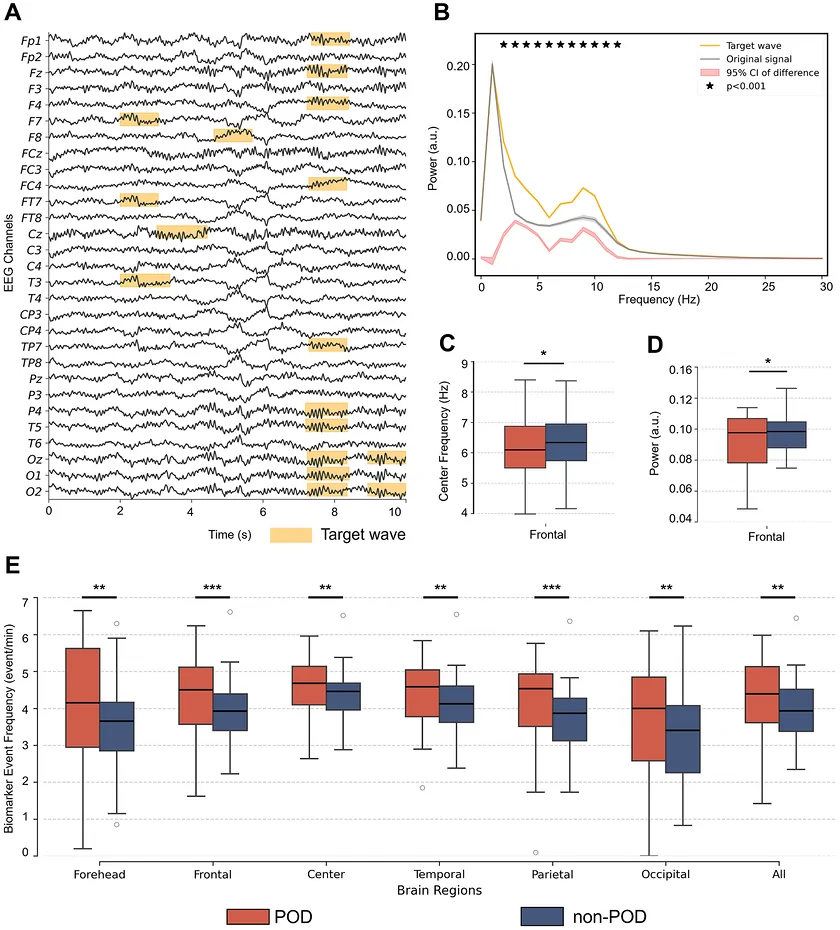
Identified EEG target wave as a potential biomarker for POD prediction in a continuous 10‐min segment. (A) Spatiotemporal distribution of the identified EEG target wave within a continuous 10‐s EEG recording. (B) Normalized PSD of the target wave compared to the original EEG signal. The differences between the PSD of the target wave and the original EEG signal are marked in pink. (C) Significant differences in the central frequency of the target wave in the frontal region between POD and non‐POD participants at the individual level. (D) Significant differences in the power of the target wave in the frontal region between POD and non‐POD participants at the individual level. (E) Statistical analysis of the average occurrence rate of the target wave per minute across different brain regions for POD and non‐POD participants. In various brain regions and across the whole brain, the frequency of biomarker events detected in POD participants was significantly higher than that in non‐POD participants. In the figure, yellow represents the target wave, gray represents the original signal, blue corresponds to POD data, and orange corresponds to non‐POD data. ***p < 0.001, **p < 0.01, *p < 0.05, based on bootstrap analysis (1000 resamples) with 99.9%, 99%, and 95% confidence intervals, respectively.

For each participant, a continuous 10‐min EEG segment was selected, ensuring no overlap with the model training set. Based on the BTF activation response intensities, target waves were identified and localized within each segment. Our result illustrates an example of a target wave (highlighted in yellow) detected in a 10‐s EEG recording from subject 008 (Figure 4A). These target waves exhibited whole‐brain synchrony and displayed spindle‐like morphology in the time domain. We further examined the frequency‐domain characteristics of these waves by comparing their power spectral density (PSD) with that of the entire 10‐min EEG segment. Statistical significance was assessed at each frequency point using bootstrap analysis (1000 resamples; 99.9% confidence interval), revealing significant differences between POD and non‐POD participants within the entire continuous 2–12 Hz range (capturing the combined δ, θ, and α activity; *p* < 0.001; Figure 4B).

To evaluate whether target waves could serve as event‐related biomarkers for POD, we compared their occurrence frequency between the POD and non‐POD groups. In the frontal region, target waves in the non‐POD group exhibited both a higher center frequency (6.38 ± 0.16 Hz vs. 6.12 ± 0.22 Hz, *p* < 0.05) and greater power (111.72 × 10^−3^ ± 3.37 × 10^−3^ vs. 111.18 × 10^−3^ ± 1.73 × 10^−3^, *p* < 0.05), as assessed by bootstrap analysis (1000 resamples; 95% confidence interval; Figure 4C,D). Of note, the target waves occurred significantly more frequently in POD participants across all individual brain regions and at the whole‐brain level (Figure 4E). Particularly prominent differences were observed in the frontal (4.62 ± 0.15 vs. 3.88 ± 0.13 waves/min, *p* < 0.001) and parietal regions (4.16 ± 0.25 vs. 3.74 ± 0.13 waves/min, *p* < 0.001), both based on bootstrap analysis with 1000 resamples and a 99.9% confidence interval. Other regions also showed significant differences at *p* < 0.01 (bootstrap analysis, 1000 resamples, 99% confidence interval). We further examined the properties of target waves in POD versus non‐POD participants (Figure S5). The variation of center frequency and power of target waves were also restricted in the frontal area. These findings suggest that EEG signals from POD and non‐POD participants elicit differential responses to the BTF, indicating that target waves, especially in the frontal cortical subregion, may represent a promising biomarker for POD prediction.

## 3 Discussion

In this study, we systematically analyzed intraoperative 32‐channel EEG signals from 71 elderly patients undergoing noncardiac surgery under anesthesia and developed an end‐to‐end POD prediction model, achieving an accuracy of 97.52% and an ROC of 0.996. Using the ST‐CN framework, we identified a characteristic EEG event strongly associated with POD incidence, manifested as a broad‐band coupled oscillatory activity spanning the 2–12 Hz (δ–θ–α) range entrained within spindle‐like envelope waveforms. This pattern occurred significantly more frequently in the intraoperative EEG of POD individuals and was predominantly localized in the frontal cortex. We further compared the POD prediction accuracy of the deep learning framework under different EEG regional input configurations and found that using EEG signals solely from the frontal region achieved a prediction accuracy of 85.1%. These results demonstrate that the BTF derived from the ST‐CN framework can serve as a novel electrophysiological signature decoder for early warning of POD.

Current risk assessment for POD in elderly patients primarily relies on clinical scales such as MMSE and CAM‐ICU, along with inflammatory biomarkers [17, 18]. These approaches exhibit substantial subjectivity, susceptibility to individual variability, and measurement heterogeneity, resulting in difficulties of intraoperative POD early warning. Although previous studies have attempted POD prediction using traditional machine learning methods such as support vector machines combined with spectral features [19, 20, 21], these approaches remain constrained by manual feature engineering limitations and poor model interpretability, resulting in limited clinical utility. While EEG‐derived metrics including the burst suppression ratio (BSR) and PSD have demonstrated promising clinical potential for POD prediction [22, 23], conventional EEG analysis methods are typically confined to predefined frequency bands such as the α/θ power ratio [24], failing to capture cross‐frequency coupling and neural dynamic temporal characteristics, which leads to critical information loss and suboptimal predictive performance.

To address the dependency on manually preset frequency bands in EEG signal analysis, we developed a POD early warning model based on the ST‐CN framework. This model employs a three‐layer network architecture that hierarchically extracts multiscale spatiotemporal features directly from raw EEG signals, enabling end‐to‐end mapping from signal to diagnosis while significantly reducing human bias and improving model objectivity and consistency. Recent applications of ST‐CN‐based EEG analysis have demonstrated success in research paradigms such as emotion recognition [12, 25]. The temporally adaptive filters learned through our ST‐CN framework effectively captured a specific band oscillations strongly associated with POD, while simultaneously exhibiting the capability to integrate multichannel coupling information in the spatial domain. Compared to traditional fixed‐band PSD analysis, our model demonstrates superior dynamic temporal modeling capabilities that better align with the complex neural mechanisms underlying POD, thereby achieving more objective and accurate characterization of POD‐specific EEG features.

The BTF extracted in this study demonstrated excellent generalization performance at both individual and population levels. Even with limited training samples, BTF‐based features combined with simple classifiers achieved stable POD discrimination, overcoming the limited generalization capability of previous machine learning methods. Analysis of 10‐min continuous EEG recordings further revealed significant differences in the occurrence frequency, central frequency, and power of target waves in the frontal region between POD and non‐POD groups. Notably, classification performance based solely on frontal signals was comparable to that using whole‐brain data. This finding aligns with the frontoparietal network abnormality theory proposed by Van Der Kooi et al., highlighting the potential of the prefrontal cortex as a key target for POD electrophysiological monitoring and providing empirical support for simplified clinical monitoring protocols [11]. The frontal and prefrontal regions, as critical hubs of the default mode network, exhibit high sensitivity to anesthesia‐induced neural oscillatory changes. Their central roles in cognitive control, emotion regulation, and attention modulation also establish them as important anatomical substrates for POD pathogenesis [26, 27, 28, 29]. Given that frontal monitoring represents the most widely adopted platform for intraoperative brain function monitoring, this strategy demonstrates significant translational potential.

Furthermore, we identified a novel electrophysiological signature using the BTF, referred to as the target wave. This signature exhibits a spindle‐like morphology in the time domain and 2–12 Hz (δ–θ–α) oscillatory coupling in the frequency domain. Considerable evidence has shown that the reciprocal interactions between the thalamic reticular nucleus (TRN) and cortical pyramidal neurons generate coherent spindle oscillations (11–16 Hz) to facilitate sleep stability [30]. Unlike healthy sleep spindles, the “target waves” identified here exhibit a pathologically lower central frequency (shifting toward θ–α range), suggesting a decompensated neural integration in thalamocortical neural network [31, 32] and the disruption of TRN‐mediated inhibitory gating driven by general anesthesia and perioperative stressors [33, 34]. Previous studies have shown that anesthesia‐induced loss of consciousness differentially disrupts α coherence between sensory thalamic nuclei and posterior cortex while abnormally enhancing α coherence between cognitive thalamic nuclei and prefrontal cortex, thereby driving an anterior shift of α rhythm [35]. The marked reduction in frontal EEG δ‐dominant low‐frequency power and relative α power ratio after such anesthesia represents a key electrographic signature of POD [9, 15]. Notably, pathological δ–α cross‐frequency coupling events can be observed in patients’ REM sleep prior to delirium onset [36]. These findings suggest that the pathological δ–α coupling unmasked by general anesthesia may reflect the neural substrate of pre‐existing cross‐frequency information integration deficits and thalamocortical rhythm dysregulation in susceptible individuals, leading to mismatched sensory and cognitive modulation and delirium phenotypes upon emergence from anesthesia.

Our study has several limitations that should be acknowledged. First, although the proposed predictive model was further evaluated using an independent external dataset and demonstrated consistently favorable performance, the external validation was conducted on a limited cohort. Therefore, additional multicenter and large‐scale prospective studies are still warranted to more comprehensively assess the generalizability and clinical applicability of the model across diverse patient populations and clinical settings. Second, the reported performance applies to intraoperative EEG segments that are either free from severe electrocautery contamination or can be effectively cleaned. In settings with frequent or intense electrocautery use, model performance may degrade unless dedicated artifact suppression methods are employed. Moreover, it is noteworthy that EEG activity during early postoperative awakening and recovery may contain valuable information related to the occurrence of POD. Future development of POD early warning models may benefit from incorporating EEG signals from different perioperative phases, thereby facilitating the construction of a more comprehensive integrated early warning framework spanning the preoperative, intraoperative, and postoperative periods.

Here, we developed a ST‐CN deep learning framework based on intraoperative multichannel EEG recording, achieving accurate prediction of high‐risk POD patients. The 2–12 Hz coupled target wave provides an interpretable novel EEG signature for POD early warning, while also clarifying the clinical value of the frontal lobe as a key monitoring target.

## 4 Materials and Methods

### 4.1 Enrollment Criteria

Patients were assessed for eligibility 1 day prior to surgery. The inclusion criteria were: (1) age ≥ 60 years; (2) American Society of Anesthesiologists (ASA) physical status classification of I–III; (3) anticipated surgical duration ≥ 120 min; and (4) voluntary participation in this clinical study. From December 2022 to October 2023, a total of 211 patients scheduled for noncardiac surgery at Xijing Hospital with an expected length of hospital stay ≥ 3 days were assessed for eligibility. Exclusion criteria included: non‐native Chinese speakers or those with speech, visual, or hearing impairments; ≤ 6 years of formal education; cognitive impairment prior to surgery (Mini‐Mental State Examination [MMSE] score ≤ 24); severe hepatic or renal insufficiency (Child–Pugh score > 5). All enrolled patients had no history of psychiatric or neurological disorders, had not used sedatives or analgesics, had no history of substance abuse, and had no contraindications to sedation or analgesia.

### 4.2 Anesthetic Procedure

Upon admission to the operating room, all patients were fitted with an EEG cap connected to a wireless multichannel EEG acquisition system for continuous real‐time signal monitoring. Routine physiological monitoring (noninvasive blood pressure [NIBP], pulse oxygen saturation [SpO_2_], electrocardiogram [ECG]) was simultaneously initiated, followed by the establishment of peripheral venous access.

Anesthesia induction was performed with intravenous propofol (1–2 mg/kg) or etomidate (0.1–0.15 mg/kg) for sedation, sufentanil (0.3–0.4 µg/kg) for analgesia, and rocuronium (0.6–0.8 mg/kg) or cisatracurium (0.15 mg/kg) for neuromuscular relaxation. Tracheal intubation was performed once the target anesthetic depth was achieved, followed by radial artery catheterization for continuous invasive arterial blood pressure monitoring.

Anesthesia maintenance was achieved with sevoflurane at 0.8–1.2 minimum alveolar concentration (MAC), combined with a continuous intravenous infusion of remifentanil (0.1–0.3 µg/kg/min). Neuromuscular relaxants were administered intermittently as clinically indicated. Intraoperatively, the inhaled concentration of sevoflurane and the infusion rate of remifentanil were titrated based on real‐time EEG monitoring, hemodynamic stability, and surgical stimulus intensity to maintain a consistent anesthetic depth throughout the procedure.

Volume‐controlled mechanical ventilation was applied with a tidal volume of 6–8 mL/kg, positive end‐expiratory pressure (PEEP) of 6 cmH_2_O, and airway pressure maintained < 30 cmH_2_O. End‐tidal carbon dioxide partial pressure (PETCO_2_) and intraoperative fluid balance were continuously monitored and optimized as needed. Blood products were administered per institutional guidelines according to the extent of intraoperative blood loss.

For antiemetic prophylaxis, intravenous ondansetron (4 mg) or palonosetron (0.25 mg) was administered 30 min prior to the conclusion of surgery. Notably, haloperidol and droperidol were strictly contraindicated for all patients in this study.

All anesthetic agents were discontinued promptly before the end of surgery. Tracheal extubation was performed only after patients had fully recovered spontaneous breathing, an effective cough reflex, stable circulatory function, and the ability to open their eyes to verbal commands. Prior to transfer to the postanesthesia care unit (PACU), all patients underwent assessment using the Steward Recovery Scale, and only those with a score of ≥ 5 were eligible for transfer.

### 4.3 EEG Acquisition and Preprocessing

During surgery, 32 scalp EEG electrodes were placed according to the international 10–20 system, with Cpz used as the reference electrode and the ground electrode placed on the forehead. The EEG data were sampled at 500 Hz, band‐pass filtered at 0.3–50 Hz, and subjected to a 50 Hz notch filter. Electrode impedances were maintained below 5 kΩ.

Segments exhibiting electrocautery interference or severe electrode dropout were excluded from the continuous intraoperative EEG recordings. Independent component analysis (ICA) was applied to reduce non‐neural artifacts; specifically, the first three independent components (ICs) associated with line noise and channel noise were removed. However, electrocautery contamination often persisted after ICA, and such segments were therefore removed to avoid losing meaningful physiological information. For electrode dropout, if fewer than four electrodes were affected, interpolation from adjacent electrodes was performed; if four or more electrodes were affected, the subject was excluded. The resulting artifact‐free EEG data were required to exceed 30 min in total duration, which was further verified by a clinician. Ultimately, data from 71 patients (27 with POD, 44 without POD) met these criteria. The selected EEG segments were then re‐referenced to the average reference and downsampled to 250 Hz.

For the ST‐CN model, the continuous EEG recordings were segmented into 10‐s epochs for training and validation. The 10‐min clean EEG segments used for biomarker extraction were selected as continuous artifact‐free segments from the intraoperative period, centered around the midpoint of the surgical procedure (i.e., approximately 50% of the total surgical duration).

### 4.4 Postoperative Delirium Screening

The primary outcome was the incidence of POD. All patients were evaluated at 1 h after extubating and on postoperative Days 1–3 (whichever came first). Each day, delirium was assessed twice (5:00–11:00 and 18:00–20:00) using the 3‐Minute Diagnostic Interview for Confusion Assessment Method‐Defined Delirium (3D‐CAM). For patients admitted to the ICU, the Confusion Assessment Method for the ICU (CAM‐ICU) was used. All evaluators were certified in the use of 3D‐CAM or CAM‐ICU and were blinded to the exposure variables. POD was defined by a positive result on either 3D‐CAM or CAM‐ICU, regardless of the number of positive assessments.

### 4.5 Spatiotemporal Convolutional Network

The proposed ST‐CN adopts a hierarchical feature extraction framework designed to analyze intraoperative EEG data for the identification of POD. The architecture initiates with a spatial convolutional layer that employs 10 two‐dimensional kernels (29 × 10 dimensions, corresponding to 29 EEG channels) to model interchannel functional connectivity patterns. The spatially encoded signals are subsequently processed through temporal convolutional layers containing 10 one‐dimensional kernels, capturing the dynamic neural oscillations over time. Each convolutional stage is followed by ReLU activation functions and average pooling, which aids in dimensionality reduction. The resulting flattened feature vectors are then passed through two fully connected layers for binary classification, distinguishing between POD and non‐POD groups.

To assess model robustness and ensure reliable performance, a fourfold cross‐validation approach was implemented, using a 75% training and 25% testing split per fold. In this model, the temporal convolution kernel and spatial convolution kernel can be conceptually regarded as a temporal filter and a spatial filter, respectively. Therefore, in subsequent descriptions, we refer to the temporal convolution kernel as the temporal filter and the spatial convolution kernel as the spatial filter for clarity and intuitive understanding.

### 4.6 Filter Analysis

To quantify the relative importance of individual spatiotemporal filters and to ensure that subsequent interpretability analyses were conducted under a well‐validated temporal parameterization, we first optimized the temporal filter length by training the model with kernel lengths of 0.5 s, 1 s, 1.5 s, and 2 s and comparing predictive performance across these settings. Model performance exhibited a marked drop at 0.5 s, whereas no significant differences were observed among 1–2 s; therefore, a temporal filter length of 1 s was selected for all subsequent analyses (Figure S6A).

With the temporal filter length fixed, we then performed a systematic contribution analysis using an iterative masking strategy. Specifically, each learned filter was zeroed out in turn while all remaining parameters were kept unchanged, and the corresponding change in model performance was recorded as the effect of removing that filter. This masking procedure was repeated across 10 independent runs, and the mean performance decrement was used as the contribution score for ranking spatial and temporal filters.

After identifying the most influential temporal filter, we further validated its stability and predictive utility and refer to this filter as the BTF. To assess robustness, we repeated model training with 20 different random initializations and observed that the highest contribution temporal filter consistently exhibited similar spectral characteristics across runs (Figure S6B–E), whereas the top‐ranked spatial filters did not show consistent spatial patterns (Figure S7), supporting a temporally specific mechanism underlying POD prediction. To validate that the filtered outputs derived from the BTF were predictive beyond the original network classifier, we additionally implemented an independent decoding pipeline in which raw EEG signals were convolved with the BTF to obtain BTF‐derived features, followed by dimensionality reduction using principal component analysis (retaining the top 10 components), and classification using a random forest classifier. We further evaluated gradient boosting and multilayer perceptron classifiers under varying training sample sizes to assess the generalizability of the BTF‐derived representations.

### 4.7 Extraction of EEG Biomarkers

The biomarker was derived from the activation response of EEG signals to the BTF. Specifically, EEG data segments of 20 s with 50% overlap were analyzed. Within each segment, the BTF convolution kernel was applied across all EEG channels, generating an activation response that reflected the degree to which the temporal dynamics of the signals aligned with the spectral characteristics of the BTF.

To detect meaningful activation patterns, we used a sliding time window (denoted as ω) with a length of 1 s, consistent with the temporal filter length. The objective was to determine whether the activation response within the sliding window exceeded a predefined threshold. The activation response was fitted to a *Gaussian* distribution (Figure S8). This threshold was defined as the mean activation response across the entire EEG segment α, plus or minus three standard deviations (mean ± 3 × std). Any activation response that exceeded this threshold was considered a statistically significant outlier, indicating an unusually strong response to the BTF.

For each sliding window ω, we counted the number of time points where the activation response exceeded the threshold. We defined this number as *τ*, with *τ* = 8 serving as the criterion for a valid response. If the activation response in a sliding window ω exceeded the threshold for at least *τ* time points, this window was marked as significant. After identifying all such marked windows, we merged them to form a continuous target wave. This target wave represented the temporal segments of the EEG that exhibited a strong response to the BTF, which we interpreted as a potential biomarker for distinguishing between different conditions. By capturing these continuous target waves, we aimed to identify patterns in the EEG that were closely associated with the presence of POD or non‐POD individuals.

### 4.8 Evaluation of Model Performance

The outcomes of the model were quantified using a set of standard classification metrics derived from the confusion matrix, including accuracy, precision, recall, and F1‐score. The mathematical equations for each metric are provided below:

(1) $$\mathrm{Accuracy}=\frac{\mathrm{TP}\;+\;\mathrm{TN}}{\mathrm{TP}\;+\;\mathrm{TN}\;+\;\mathrm{FP}\;+\;\mathrm{FN}}$$

(2) $$\mathrm{Precision}=\frac{\mathrm{TP}}{\mathrm{TP}\;+\;\mathrm{FP}}$$

(3) $$\mathrm{Recall}=\frac{\;\mathrm{TP}}{\mathrm{TP}\;+\;\mathrm{FN}}\;$$

(4) $$F1-\mathrm{score}\;=\frac{2\;\times \;\mathrm{Pre}\;\times \;\mathrm{Rec}}{\mathrm{Pre}\;+\;\mathrm{Rec}}$$

Diagnostic capability was further characterized by receiver operating characteristic (ROC) curve analysis, with area under the curve (AUC) values summarizing overall classification performance. These metrics collectively evaluate the discriminative capacity of the ST‐CN for distinguishing between POD and non‐POD groups.

### 4.9 Statistical Analyses

In this study, statistical analysis was performed using bootstrapping with *Bonferroni* correction. Bootstrapping was conducted with 1000 iterations, and the *Bonferroni* correction was applied to adjust the significance threshold for multiple comparisons. Normally distributed continuous variables are expressed as means ± standard deviations, whereas non‐normally distributed continuous variables are reported as medians with interquartile ranges (25th to 75th percentiles). All analyses were carried out using Python 3.10.

## Author Contributions

Yinuo Zhang, Yan Zhu, and Xinxin Zhang performed study design, data analysis, result interpretation, and manuscript drafting and revision. Xinke Shen contributed to methodology through model design and development. Xuemiao Tang, Zhihong Lu, Chong Lei, and Mengyu Li performed data analysis and sample collection. Hailong Dong supervised sample collection and reviewed the manuscript. Guangchao Zhao, Quanying Liu, and Zhichao Liang conceived the study, acquired funding, supervised the entire research process, and critically revised the manuscript. All the authors have read and approved the final manuscript.

## Funding

This work was supported by the National Science and Technology Major Project (2021ZD0200500, 2025ZD0218300); National Natural Science Foundation of China (3254100307, 62472206, 82271211, 82293643, 82430040, 82221001); the Young Elite Scientist Sponsorship Program by CAST (No. YESS20220212); Shenzhen Science and Technology Innovation Committee (RCYX20231211090405003, KJZD20230923115221044); Guangdong Basic and Applied Basic Research Foundation (2025A1515011645, 2026A1515010121, 2026B1515020099); Guangdong S&T Program (2026B0101110003); Shanghai Municipal Special Program for Basic Research on General AI Foundation Models (2025SHZDZX026D05); Shenzhen Doctoral Startup Project (RCBS20231211090748082); the open research fund of the Guangdong Provincial Key Laboratory of Mathematical and Neural Dynamical Systems; the Center for Computational Science and Engineering at Southern University of Science and Technology, Shenzhen Key Laboratory of Smart Healthcare Engineering; the Joint Founding Project of Innovation Research Institute, Xijing Hospital (No. LHJJ24JH02).

## Ethics Statement

The primary cohort study protocol was approved by the Institutional Review Board of Xijing Hospital (Approval Number: KY2022213‐C‐1) and registered at the Chinese Clinical Trial Registry (ChiCTR2200066376) prior to patient enrollment. The independent external validation dataset was separately approved by the Ethics Committee of Xijing Hospital (Approval Number: KY20232361‐C‐1) and registered with ClinicalTrials.gov (Identifier: NCT06161662). All procedures were carried out in accordance with the Code of Ethics of the World Medical Association (Declaration of Helsinki) for experiments involving humans. Written informed consent was obtained from all participants in both cohorts.

## Conflicts of Interest

The authors declare no conflicts of interest.

## Supporting information




**Supporting File 1**: mco270980‐sup‐0001‐SuppMat.docx

## Data Availability

The analysis code has been uploaded to GitHub, the code is available at https://github.com/ncclab‐sustech/POD‐biomarker.git. Detailed experimental protocols and analysis code are provided in Section 4 Materials and Methods. The data that support the findings of this study are available upon reasonable request from the corresponding author.
