# Supplementary material for: An Intraoperative EEG Biomarker for Postoperative Delirium Predicting Based on Interpretable Deep Learning Framework
Source: MedComm (2020). 2026 Sep 6;7(9):e70980. doi: 10.1002/mco2.70980 (PMC13547085; doi:10.1002/mco2.70980)
Supplement: Supplementary file 1 [file MCO2-7-e70980-s001.docx]

**An intraoperative EEG biomarker for postoperative delirium predicting based on interpretable deep learning framework**

Yinuo Zhang,^1,†^ Yan Zhu,^1,†^ Xinxin Zhang,^2,3,4,†^ Xinke Shen,^1^ Xuemiao Tang^2,5^, Zhihong Lu^2,3,4^, Chong Lei^2,3,4^, Mengyu Li^2,3,4^, Hailong Dong^2,3,4^, Zhichao Liang,^1,6,*^ Quanying Liu,^1,*^ and Guangchao Zhao^2,3,4,*^

1 Department of Biomedical Engineering, Southern University of Science and Technology, Shenzhen, Guangdong, China

2 Department of Anesthesiology and Perioperative Medicine, Xijing Hospital, The Fourth Military Medical University, Xi’an, China

3 Key Laboratory of Anesthesiology (The Fourth Military Medical University), Ministry of Education, Xi’an, China

4 Shaanxi Provincial Clinical Research Center for Anesthesiology Medicine, Xi’an, China

5 Department of Anesthesiology, The Third People’s Hospital of Chengdu, Chengdu, Sichuan, China

6 Center for Neurocognition and Social Behavior, Artificial Intelligence Research Institute, Shenzhen University of Advanced Technology, Shenzhen, Guangdong, China

^†^These authors contributed equally to this work.

*Correspondence to: Zhichao Liang, Quanying Liu, Guangchao Zhao

Full address: Department of Anesthesiology and Perioperative Medicine, Xijing hospital, Changle West Road, Xi’an, Shaan’xi, 710032, China.

E-mail: [liangzc@sustech.edu.cn](mailto:liangzc@sustech.edu.cn); [liuqy@sustech.edu.cn](mailto:liuqy@sustech.edu.cn); [gczhao0518@hotmail.com](mailto:gczhao0518@hotmail.com)

# Supplementary Data

**Figure S1. Analysis of model results in the first fold. A.** Ten spatial filters visualized on the scalp topography; red indicates activation and blue indicates suppression. **B.** Ten temporal filters plotted over time; each window captures 1 s of information. **C.** Classification performance when each temporal filter is used independently as a feature extractor. **D.** Classification performance when each brain region is used independently for classification. **E.** Model classification performance under different training-set proportions. **F.** Subject-level diagnosis prediction (whether the subject is diagnosed with the disease).

**Figure S2. Analysis of model results in the second fold. A.** Ten spatial filters visualized on the scalp topography; red indicates activation and blue indicates suppression. **B.** Ten temporal filters plotted over time; each window captures 1 s of information. **C.** Classification performance when each temporal filter is used independently as a feature extractor. **D.** Classification performance when each brain region is used independently for classification. **E.** Model classification performance under different training-set proportions. **F.** Subject-level diagnosis prediction (whether the subject is diagnosed with the disease).

**Figure S3. Analysis of model results in the third fold. A.** Ten spatial filters visualized on the scalp topography; red indicates activation and blue indicates suppression. **B.** Ten temporal filters plotted over time; each window captures 1 s of information. **C.** Classification performance when each temporal filter is used independently as a feature extractor. **D.** Classification performance when each brain region is used independently for classification. **E.** Model classification performance under different training-set proportions. **F.** Subject-level diagnosis prediction (whether the subject is diagnosed with the disease).

**Figure S4.** **Classification accuracy by different frontal groups.** **A.** Diagram of EEG electrode positions and corresponding frontal electrode configurations for the three groups. **B.** Classification accuracy by different frontal groups.

**Figure S5. Comparison of the power and center frequency of the target wave between POD and non-POD across brain regions. A.** Power comparison of the target wave between POD and non-POD across brain regions. **B.** Center-frequency comparison of the target wave between POD and non-POD across brain regions. * denotes p < 0.05 (bootstrap, 1,000 resamples; 95% confidence interval).

**Figure S6. Comparison of spatiotemporal models with different temporal filter lengths. A.** Classification performance of models with temporal filter lengths of 0.5 s, 1.0 s, 1.5 s, and 2.0 s. For each length, 20 models were trained from random initializations, and the best-performing model from a four-fold cross-validation was selected for comparison. Performance was significantly worse for the 0.5 s temporal filter length compared with the others (Welch’s t-test, ***p < 0.001). No significant differences were observed among the other filter lengths. **B–E**. Power spectral density (PSD) of the optimal temporal filter for lengths of 0.5 s (B), 1.0 s (C), 1.5 s (D), and 2.0 s (E). Shaded areas represent the 95% confidence interval.

**Figure S7.** **Activation of high-contribution spatial filters across brain regions under different temporal filter lengths. A–D.** Activation patterns of high-contribution spatial filters (each contributing >4% to model performance) across EEG channels at temporal filter lengths of 0.5 s (A), 1.0 s (B), 1.5 s (C), and 2.0 s (D). **E–F.** Mean activation of these high-contribution spatial filters across six brain regions for each temporal filter length. For each length, 20 models were trained from random initializations, and the best-performing model from a four-fold cross-validation was used for analysis.

**Figure S8. The BTF activation in single-channel EEG follows a Gaussian distribution.**

**Table S1. The surgical types of the enrolled patients.**

| **ID** | **date** | **age** | **ASA** | **Type of surgery** |
| --- | --- | --- | --- | --- |
| H77060 | 20/10/2022 | 62 | 3 | Pancreaticoduodenectomy, Cholecystectomy, Intestinal anastomosis, Chemotherapy particle implantation |
| H78425 | 26/10/2022 | 63 | 2 | Partial bile duct resection, Biliary-enteric anastomosis, Partial hepatectomy |
| H79080 | 30/10/2022 | 65 | 2 | Liver tumor resection, Abdominal adhesiolysis |
| H80164 | 03/11/2022 | 70 | 2 | Laparoscopic pancreaticoduodenectomy |
| H79444 | 06/11/2022 | 71 | 2 | Right hemicolectomy |
| H83202 | 24/11/2022 | 69 | 3 | Cholangiocarcinoma resection, Left hemihepatectomy, Cholecystectomy, Hepatic hilar bile duct reconstruction, Abdominal lymph node dissection, Chemotherapy particle implantation |
| A31856 | 27/11/2022 | 76 | 3 | Robot-assisted laparoscopic radical prostatectomy |
| H80733 | 05/12/2022 | 68 | 2 | Robot-assisted laparoscopic radical prostatectomy |
| H84968 | 06/12/2022 | 64 | 2 | Laparoscopic radical distal gastrectomy |
| H86484 | 11/12/2022 | 71 | 2 | Laparoscopic pancreatic tail and spleen resection, Lysis of abdominal adhesions |
| 909325 | 14/12/2022 | 73 | 2 | Laparoscopic pancreatic tail and spleen resection, Hepatic nodule excision |
| H86615 | 10/01/2023 | 66 | 2 | Pancreaticoduodenectomy, Cholecystectomy, Intestinal anastomosis, Abdominal lymph node dissection |
| H87670 | 11/01/2023 | 71 | 2 | Pancreaticoduodenectomy, Intestinal-to-intestinal anastomosis, Abdominal lymph node dissection, Abdominal adhesiolysis |
| H90196 | 15/01/2023 | 69 | 2 | Pancreaticoduodenectomy, Cholecystectomy, Intestinal-to-intestinal anastomosis, Abdominal lymph node dissection |
| H94304 | 14/02/2023 | 65 | 2 | Laparoscopic total gastrectomy, Abdominal lymph node dissection |
| H93689 | 16/02/2023 | 71 | 2 | Laparoscopic pancreaticoduodenectomy, Cholecystectomy |
| H99958 | 02/03/2023 | 76 | 2 | Laparoscopic radical resection for colorectal cancer, Chemotherapy particle implantation |
| E60019 | 07/03/2023 | 62 | 2 | Laparoscopic left hepatic hepatectomy, Cholecystectomy |
| F32437 | 20/03/2023 | 61 | 2 | Robot-assisted laparoscopic radical prostatectomy |
| I04403 | 20/03/2023 | 71 | 3 | Robot-assisted retroperitoneal laparoscopic partial nephrectomy of the left kidney |
| F45345 | 28/03/2023 | 66 | 2 | Robot-assisted laparoscopic radical prostatectomy |
| I06025 | 28/03/2023 | 75 | 2 | Cholecystectomy, Choledochoscopy with biliary exploration and stone removal, T-tube drainage, Lysis of intraperitoneal adhesions |
| F75635 | 29/03/2023 | 68 | 2 | Robot-assisted laparoscopic radical prostatectomy |
| H83344 | 30/03/2023 | 66 | 2 | Robot-assisted laparoscopic pelvic lymph node dissection, Right inguinal lymph node dissection |
| F40394 | 09/04/2023 | 66 | 2 | Robot-assisted laparoscopic radical prostatectomy |
| I09909 | 09/04/2023 | 62 | 2 | Posterior laparoscopic radical nephrectomy for left renal cell carcinoma |
| I08014 | 12/04/2023 | 65 | 3 | Laparoscopic pancreaticoduodenectomy, Cholecystectomy, Intestinal anastomosis, Chemotherapy particle implantation |
| F57189 | 18/04/2023 | 66 | 3 | Laparoscopic radical cystectomy, Ileal in situ neocystoplasty, Appendectomy, Pelvic lymph node dissection |
| I13332 | 23/04/2023 | 68 | 2 | Pancreaticoduodenectomy, Cholecystectomy, Intestinal anastomosis, Abdominal lymph node dissection, Chemotherapy particle implantation |
| I16853 | 08/05/2023 | 60 | 2 | Partial gastrectomy, Liver tissue biopsy, Cholecystectomy, Gastroenterostomy, Intestinal anastomosis |
| I10128 | 09/05/2023 | 66 | 2 | Robot-assisted laparoscopic radical prostatectomy, Bilateral pelvic lymph node dissection |
| I17377 | 09/05/2023 | 70 | 2 | Radical cholecystectomy |
| I09102 | 10/05/2023 | 65 | 2 | Robot-assisted laparoscopic radical prostatectomy |
| E80543 | 10/05/2023 | 66 | 2 | Robot-assisted laparoscopic radical prostatectomy, Bilateral pelvic lymph node dissection |
| I18911 | 16/05/2023 | 68 | 2 | Laparoscopic total gastrectomy, Intestinal-to-intestinal anastomosis, Abdominal lymph node dissection, Chemotherapy particle implantation |
| I19446 | 25/05/2023 | 74 | 2 | Laparoscopic distal gastrectomy, Abdominal lymph node dissection, Intestinal-intestinal anastomosis |
| I20356 | 28/05/2023 | 75 | 2 | Pancreatic body and tail resection with splenectomy, Left adrenalectomy, Abdominal lymph node dissection, Liver cyst fenestration with drainage |
| I09052 | 31/05/2023 | 70 | 2 | Robot-assisted laparoscopic radical prostatectomy, Bilateral pelvic lymph node dissection |
| I24557 | 12/06/2023 | 64 | 2 | Robot-assisted laparoscopic partial nephrectomy of the left kidney |
| G92858 | 19/06/2023 | 63 | 2 | Robot-assisted laparoscopic radical cystectomy, Ileal bladder reconstruction |
| I24975 | 20/06/2023 | 79 | 2 | Retroperitoneal laparoscopic right nephrectomy, Right ureterectomy + partial cystectomy |
| I27399 | 20/06/2023 | 66 | 2 | Robot-assisted laparoscopic radical prostatectomy |
| I27491 | 26/06/2023 | 63 | 3 | Robot-assisted laparoscopic radical prostatectomy |
| I23530 | 28/06/2023 | 64 | 2 | Robot-assisted laparoscopic radical prostatectomy |
| H11143 | 18/10/2022 | 62 | 3 | Laparoscopic complex right hepatic hepatectomy, Cholecystectomy, Lysis of intraperitoneal adhesions, Chemotherapy particle implantation |
| H79035 | 27/10/2022 | 67 | 2 | Laparoscopic pancreaticoduodenectomy, Cholecystectomy, Abdominal lymph node dissection, Chemotherapy particle implantation |
| 907283 | 30/10/2022 | 70 | 2 | Laparoscopic pancreaticoduodenectomy, Laparoscopic adhesion lysis, Laparoscopic lymph node dissection |
| H79180 | 30/10/2022 | 67 | 2 | Pancreatic body and tail resection with splenectomy |
| H76281 | 01/11/2022 | 68 | 2 | Robot-assisted laparoscopic radical prostatectomy, Pelvic lymph node dissection |
| H80586 | 06/11/2022 | 64 | 2 | Pancreaticoduodenectomy, Intestinal-intestinal anastomosis, Extensive intra-abdominal adhesion lysis |
| 951920 | 08/01/2023 | 63 | 2 | Laparoscopic liver biopsy, Chemotherapy particle implantation |
| H90242 | 15/01/2023 | 75 | 2 | Abdominoperineal resection with radical rectal cancer surgery |
| H94745 | 13/02/2023 | 74 | 2 | Pancreatic body and tail resection with splenectomy |
| H99494 | 28/02/2023 | 69 | 3 | Extensive intra-abdominal adhesion lysis, Partial hepatectomy, Choledochoscopy with stone extraction, T-tube drainage, Chemotherapy particle implantation |
| A92206 | 08/03/2023 | 69 | 2 | Laparoscopic spleen-sparing pancreatic body-tail resection |
| F30379 | 09/04/2023 | 68 | 3 | Robot-assisted laparoscopic right nephrectomy, Right ureterectomy, Radical cystectomy, Left ureterostomy |
| I05552 | 10/04/2023 | 63 | 2 | Robot-assisted laparoscopic radical prostatectomy, Bilateral pelvic lymph node dissection |
| I09603 | 11/04/2023 | 61 | 2 | Pancreatic body and tail resection with splenectomy, Right nephrectomy, Cholecystectomy |
| I09162 | 13/04/2023 | 71 | 2 | Laparoscopic pancreaticoduodenectomy, Cholecystectomy, Chemotherapy particle implantation |
| I10581 | 17/04/2023 | 62 | 3 | Laparoscopic spleen-sparing pancreatic body-tail resection |
| I07551 | 03/05/2023 | 62 | 2 | Pancreaticoduodenectomy, Cholecystectomy, Intestinal-intestinal anastomosis, Laparoscopic lymph node dissection |
| D14941 | 04/05/2023 | 71 | 2 | Posterior laparoscopic partial nephrectomy of the left kidney |
| D86073 | 07/05/2023 | 85 | 3 | Robot-assisted laparoscopic radical prostatectomy |
| I18423 | 14/05/2023 | 65 | 2 | Abdominal nodule biopsy, Cholecystectomy, Cholangioenterostomy, Gastrointestinal anastomosis, Intestinal-intestinal anastomosis |
| I20737 | 24/05/2023 | 61 | 2 | Robot-assisted laparoscopic partial nephrectomy of the left kidney |
| I21292 | 29/05/2023 | 73 | 2 | Robot-assisted laparoscopic radical prostatectomy, Bilateral pelvic lymph node dissection |
| I22404 | 29/05/2023 | 69 | 2 | Robot-assisted laparoscopic radical prostatectomy |
| E88502 | 05/06/2023 | 63 | 3 | Robot-assisted laparoscopic radical prostatectomy |
| A39640 | 07/06/2023 | 78 | 2 | Pancreaticoduodenectomy, Cholecystectomy, Intestinal-intestinal anastomosis, Abdominal lymph node dissection |
| H21782 | 11/06/2023 | 74 | 2 | Laparoscopic splenectomy |
| I26416 | 18/06/2023 | 64 | 2 | Robot-assisted laparoscopic partial nephrectomy of the left kidney |

The table details the ID, surgery date (day/month/year), age, ASA classification, and specific types of surgeries of the enrolled patients, including but not limited to pancreatic surgery, hepatobiliary surgery, gastrointestinal surgery, urological surgery, etc.
